# *Armillifer* Infections in Humans: A Systematic Review

**DOI:** 10.3390/tropicalmed4020080

**Published:** 2019-05-16

**Authors:** Petros Ioannou, Rodanthi Vamvoukaki

**Affiliations:** Department of Internal Medicine & Infectious Diseases, University Hospital of Heraklion, Heraklion, PC71303 Crete, Greece; rodoulavamv@gmail.com

**Keywords:** *Armillifer*, parasite, tropical diseases

## Abstract

*Armillifer* is a genus of obligate parasites belonging to the Pentastomida subclass that was first described as a cause of human disease in 1847. Human infection by *Armillifer* is rare and not widely known. These parasites are transmitted to humans by handling or eating undercooked meat from infected snakes, which are the definitive hosts, or oral uptake of environmental ova. The aim of this systematic review was to record all available evidence regarding infections by *Armillifer* in humans. A systematic review of PubMed (through 21 December 2018) for studies providing epidemiological, clinical, microbiological, as well as treatment data and outcomes of *Armillifer* infections was conducted. A total of 26 studies, containing data of 40 patients, were eventually included in the analysis. The most common sites of infection were the peritoneal cavity, the liver, the lower respiratory and the abdominal tract. The commonest infecting species was *A. armillatus* and most patients were asymptomatic; however, when symptoms occurred, the commonest was abdominal pain, even though unusual presentations occurred, such as hepatic encephalopathy or neurologic symptoms. Most cases were diagnosed at surgery or by imaging, and most patients were not treated. Mortality was low, but the majority of the cases with ocular infection lead to permanent loss of vision.

## 1. Introduction

*Armillifer* is a genus of obligate parasites belonging to the Pentastomida subclass that was first described as cause of human disease in 1847 [1]. The commonest *Armillifer* species are *A. armillatus, A. grandis, A. agkistrodontis, A. moniliformis,* and *A. mazzai*. Adult parasites of the *Armillifer* genus inhabit the respiratory system of snakes who are the definite hosts, and their infective ova are excreted by the snakes feces and secretions and are digested by small mammals who are the intermediate hosts. After ingestion of the infective parasite eggs, larvae migrate to various organs where they encyst. Infection of snakes then occurs by eating infected rodents [2]. Different species have slight differences in their life cycles, for example the definitive hosts of *A. armillatus* are pythons, while the definitive hosts of *A. grandis* are viperid snakes.

Human infection by *Armillifer* is rare and not widely known. *Armillifer* parasites are transmitted to humans by handling or eating undercooked meat from infected snakes, or by oral uptake of environmental ova. In the majority of cases, the parasite load is low, the patient remains asymptomatic, and the diagnosis is either made incidentally with an X-ray that shows the calcified parasites, or at autopsy, however in some cases it can be symptomatic and has been associated with severe outcomes [3,4,5].

## 2. Methods

### 2.1. Data Search

For this review, we adopted the Preferred Reporting Items for Systematic Reviews and Meta-analyses (PRISMA) guidelines [6]. Eligible studies were identified through search of PubMed MEDLINE with the following text-words: Armillif*[tw]. Day of last search was 21 December 2018.

### 2.2. Study Selection

Studies were included in the analysis if they met the following criteria: (1) Published in English; (2) Reporting data on patients’ clinical characteristics, microbiology, treatment, and outcomes. Studies with the following criteria were excluded from the analysis: (1) Secondary research papers (e.g., reviews), editorials, and papers not reporting results on primary research; (2) studies not in humans; (3) studies not in English. The titles of the resulting references were screened using Rayyan [7]. Then the full text articles were retrieved and rescreened for potentially relevant articles. Reference lists of included studies were searched for relevant articles.

### 2.3. Endpoints

The study endpoint was to record the type of *Armillifer* infections included in the literature as well as the patient characteristics for different type of infections, the clinical data on *Armillifer* infections, and their treatment and outcomes.

### 2.4. Data Extraction and Definitions

Data from each eligible study was extracted by the investigators (PI, RV). The extracted data included study type, year of publication, and country; patient demographic data (age and gender); patient’s relevant medical history; infection data, clinical data; treatment administered for the infection; and outcomes (i.e., cure or death). Relation of death to the index infection was reported according to the study authors. The complications recorded included any organ dysfunction or clinical deterioration that was considered by the authors to be related to the *Armillifer* infection.

## 3. Results

### 3.1. Literature Search

**Figure 1:**
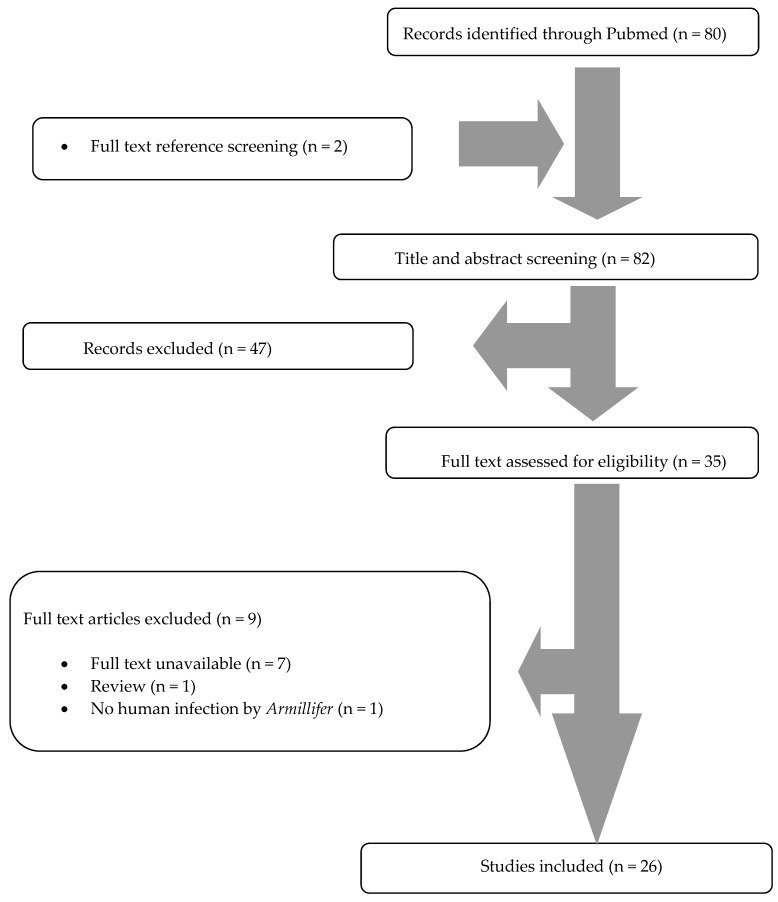
Preferred Reporting Items for Systematic Reviews and Meta-analyses (PRISMA) flow diagram.

A total of 80 articles from PubMed were screened. After reviewing the titles and abstracts, 33 articles were selected for full-text review. From them, nine were excluded: one was not an original paper (review), one did not involve infection in humans, and there were seven articles whose full text was unavailable. Two additional studies were found by hand-screening of the included articles’ references. Finally, 26 met the study criteria [3,4,8,9,10,11,12,13,14,15,16,17,18,19,20,21,22,23,24,25,26,27,28,29,30,31]. The review process is graphically presented in Figure 1.

### 3.2. Included Studies’ Characteristics

The 26 studies that were finally included in this analysis involved a total of 40 patients with 12 studies conducted in Africa, 7 in Europe, 4 in Asia, and 3 in North America. The final sum included 19 case reports and 7 case series.

### 3.3. Epidemiology, Clinical Data, Treatment, and Outcomes of Armillifer Infections

The patients’ age ranged from 3 to 80 years, with a mean age of 36.6 years; 68.4% (when data were available) were male. The commonest sites of infections were the peritoneal cavity in 70% (28 patients), liver in 50% (20 patients), lower respiratory tract in 30% (12 patients), gastrointestinal tract in 22.5% (9 patients), spleen in 17.5% (7 patients), eye in 15% (6 patients), and the kidneys, urinary bladder and genital tract in 5% (2 patients) each. The species causing the infection was *A. armillatus* in 62.5% of patients (25 patients), *A. grandis* in 17.5% (7 patients), *A. moniliformis* in 12.5% (5 patients), and *A. agkistrodontis* in 10% (4 patients), while in 20% (8 patients) the species was unknown. Among patients with available data, 63.6% (14 out of 22 patients) reported snake eating, 13.6% (3 patients) reported worm eating, and 4.5% (1 patient) reported alligator eating. Symptoms were present in 45% (18 patients), with pain being the commonest, affecting 25% (10 patients). Other symptoms included fever and sepsis in 10% (4 patients) each, cough or acute abdomen in 5% (2 patients) each, and neurologic symptoms or hepatic encephalopathy in 2.5% (1 patient) each. Diagnosis was made in surgery in 42.5% (17 patients), by imaging in 30% (12 patients), clinically in 15% (6 patients), and during autopsy in 12.5% (5 patients). When mentioned, eosinophils were increased in 42.7% (3 out of 7 patients). Among the infected patients, 25% (6 out of 24 patients) were medically treated and the regiments used were thiabendazole in 16.7% (4 patients), praziquantel in 12.5% (3 patients), mebendazole, ciprofloxacin, hematinics, and traditional Chinese medicine in 4.2% (1 patient) each. Glucocorticoids were not used in any case. Surgical management was performed in 20.8% (5 patients), while 54.2% (13 patients) were not treated. Clinical cure was achieved in 57.9% (11 out of 17 patients), while permanent loss of vision occurred in 66.7% (4 out of 6 patients) with an eye infection. Five patients died (12.5%), but death was attributed to *Armillifer* infection in 5% (2 patients). A synopsis of the studies describing human infections by *Armillifer* species is shown in Table 1. In a contingency statistical analysis model, cure was not found to be associated with multi-organ involvement, *Armillifer* subspecies infection, or snake eating. Furthermore, in a linear regression analysis model, eosinophilia was not found to be associated with the presence of calcifications or cure, and the presence of calcifications was not found to be associated with the presence of symptoms.

## 4. Discussion

Most cases reported in humans are due to *A. armillatus*, which is endemic in West and Central Africa; however, the last decades such cases have been reported in African immigrants in North America and Europe [2,13,14]. *Armillifer* infection mostly affects men and can affect any age. History of snake eating was highly prevalent in patients with *Armillifer* infection. The most common sites of infection were the peritoneal cavity, the liver, the lower respiratory and the gastrointestinal tract. Interestingly, *A. armillatus* is the commonest isolated parasite, however, pathology and molecular methods were infrequently used in order to accurately classify the parasites. Thus, since the diagnosis of the species was many times empirical, it could be that the species were misdiagnosed in some of these studies.

Importantly, only 45% of the patients were symptomatic, however, this probably is not representative of the true clinical picture of infection by *Armillifer*, since it is reasonable that most cases of asymptomatic infections are undetected and underrepresented in this systematic review. Interestingly, even though abdominal pain was the commonest symptom, symptomatic patients had developed a wide range of symptoms, like hepatic encephalopathy, neurologic symptoms, and fever. Diagnosis was set during surgery, by imaging or incidentally during autopsy in the majority of cases, which is in line with the fact that most cases were asymptomatic. Thus, it is not a surprise that medical management was chosen in only 20% of cases, however the clinical cure rate was only 57.9%. Importantly, ocular infections appeared to be devastating, carrying a very bad prognosis, with about 67% of patients permanently losing their vision. Armillifer-specific mortality was not high, but due to underrepresentation of the asymptomatic infections, it is anticipated that mortality will be even lower. Interestingly, in both cases were death was associated with the infection by *Armillifer* parasites, numerous parasites had been found at autopsy, implying that the occurrence of serious adverse events could be associated with the number of parasites in the host.

Even though it would be tempting to treat any patient with an infection by *Armillifer* parasites, the effectiveness of the anti-helminthic medications has not been established in the literature. Furthermore, given that treatment of a patient could lead to release of antigens that may lead to hypersensitivity reactions and a paradoxical worsening of the symptoms, it is not clear that treatment would be of benefit, especially for the asymptomatic patient [32]. Importantly, there are no guidelines or a consensus regarding the optimal treatment of these patients, nor there are any data on the effectiveness of the anti-helminthic medications. Thus, it is not a surprise that several medications had been used, such as thiabendazole, praziquantel, mebendazole, ciprofloxacin, hematinics, and even traditional Chinese medicine. Thus, we believe it would be important to find tools that could allow identification of the patient population that would benefit from treatment with anti-helminthics. Statistical analysis of the data presented in this review did not show that eosinophilia or the absence of calcifications could be of any use to guide treatment. Interestingly, even though eosinophilia is classically associated with parasitic infection, it was present in only about 40% of patients; however, data were available for only 7 patients, all of whom were symptomatic. Thus, even though the data do not allow drawing safe conclusions, it would be reasonable to treat symptomatic patients with an anti-helminthic medication, with the possible exception of eye infection, where anti-helminthic treatment would not be effective enough to treat the infection and improve vision, while, on the contrary, the release of antigens inside the eye could be catastrophic in terms of visual acuity.

The possibility of causing a paradoxic or an anaphylactic reaction after treatment with anti-helminthics due to release of helminthic antigens could argue for the use of glucocorticoids to reduce such a response [32]. Interestingly, glucocorticoids were not used in any study in this review. Furthermore, such a paradoxic response was not described by any of the authors. Thus, the use of glucocorticoids is not supported by the existing evidence.

The present systematic review has certain limitations that should be acknowledged. First of all, it only consists of case reports and case series, so, the results should be read with caution, as case reports are descriptions of unusual presentations, while the usual ones may be underrepresented in a systematic review consisting of such studies. However, if case reports, and case series were excluded, as other investigators have done in other cases [33], there would be no studies left for inclusion. Thus, all informative cases reliably demonstrating the nature of infections by *Armillifer* have been included in this analysis. Secondly, it could be that some cases of *Armillifer* infections had been missed during our literature search if they had used a different genus name than *Armillifer*, such as *Porocephalus*. Finally, there are many studies that have been published in languages other than English, and they have been excluded from our study.

In conclusion, medical management was chosen in one out of five cases of *Armillifer* infections, however the clinical cure was just over 50%. Ocular infections were devastating with two out of three cases leading to permanent vision loss. Mortality was low, and due to underrepresentation of the asymptomatic infections, it is anticipated to be even lower. Physicians caring for patients in endemic regions or working with returning travelers should become familiar with these infections.

## Figures and Tables

**Table 1:** Synopsis of studies describing human infections by *Armillifer* species.

Author [Ref]	Year	Species	Site of Infection	Clinical Manifestation	Diagnosed by	Treatment	Outcome
Tappe [3]	2016	7 *A. armillatus* 3 *A. grandis*	7 Peritoneum	None	7 Surgery	NR	NA
Yapo Ette [4]	2003	*A. grandis*	1 Lungs, bowel, lover, spleen, kidneys, peritoneum	Abdominal pain	Autopsy	NA	Death
Aiyekomogbon [8]	2016	3 *A. armillatus*	3 Liver, 2 peritoneum, bowel, 1 urinary bladder, kidneys, spleen, lungs	Abdominal pain, cough	3 Imaging	2 Medical 1 None	2 Cured
Potters [9]	2017	1 *A. armillatus*	1 Peritoneum	None	Surgery	None	NA
Beltram [10]	2017	1 *A. armillatus*	1 Peritoneum	Not reported	Autopsy	NA	NA
Tappe [11]	2015	1 *A. grandis* 1 *A. moniliformis* 1 *A. agkistrodontis* 1 Unknown	2 Peritoneum	None	2 Surgery	NR	NA
Sulyok [12]	2014	2 *A. grandis* 2 *A. moniliformis* 2 *A. agkistrodontis* 2 Unknown	4 Eye	4 Ocular	4 Clinically	2 Surgical 2 None	4 Lost vision
Tappe [13]	2014	1 *A. armillatus*	1 Genital tract	Abdominal pain	Surgery	Surgical	Cured
Tappe [14]	2013	1 *A. armillatus*	1 Liver	None	Autopsy	NA	NA
Ye [15]	2013	1 Unknown	1 Lungs, liver, peritoneum	Fever, abdominal pain	Surgery	Medical	Cured
Wang [16]	2013	1 *A. moniliformis* 1 *A. agkistrodontis* 1 Unknown	2 Peritoneum, liver, 1 lungs, bowel	2 Fever, 1 abdominal pain	2 Surgery	Medical	2 Cured
Jisieike-Onuigbo [17]	2011	1 *A. armillatus*	1 Lungs, liver, peritoneum	Abdominal pain	Imaging	Medical	Cured
Latif [18]	2011	1 *A. moniliformis*	1 Liver	None	Surgery	None	NA
Ibinaiye [19]	2011	1 Unknown	1 Liver, peritoneum	Acute abdomen, fever	Imaging	None	Cured
Adeyekun [20]	2011	1 *A. armillatus*	1 Liver, urinary bladder, peritoneum, spleen, lungs	Abdominal pain	Imaging	None	Not cured
Lavarde [21]	1999	1 *A. armillatus*	1 Lungs, bowel, liver, peritoneum	Neurologic symptoms	Autopsy	NA	Death
Guardia [22]	1991	1 *A. armillatus*	1 Lungs, bowel, liver, peritoneum	None	Autopsy	NA	NA
Herzog [23]	1985	1 *A. armillatus*	1 Bowel, peritoneum	Acute abdomen	Surgery	Medical	Not cured
Mapp [24]	1976	1 Unknown	1 Liver, peritoneum	None	Imaging	None	NA
Goldsmid [25]	1973	1 *A. armillatus*	1 Genital tract	Abdominal pain	Surgery	None	Cured
Lazar [26]	1967	1 *A. armillatus*	1 Eye	Ocular	Clinically	Surgical	Cured
Bretland [27]	1962	2 *A. armillatus*	2 Lungs, liver, spleen, peritoneum	None	2 Imaging	2 None	NA
Neumann [28]	1962	1 *A. armillatus*	1 Eyelid	Ocular	Clinically	Surgical	Cured
Steinbach [29]	1957	1 Unknown	1 Lungs, bowel, liver, spleen, peritoneum	None	Imaging	None	NA
du Plessis [30]	2007	1 *A. armillatus*	1 Liver	None	Imaging	None	NA
Sellier [31]	2004	1 Unknown	1 Lungs, liver, bowel, spleen, peritoneum	None	Imaging	NR	NA

NR: Not reported, NA: Not applicable.
